# iTRAQ-based quantitative proteome analysis insights into cold stress of Winter Rapeseed (*Brassica rapa* L.) grown in the field

**DOI:** 10.1038/s41598-021-02707-z

**Published:** 2021-12-06

**Authors:** Zaoxia Niu, Lijun Liu, Yuanyuan Pu, Li Ma, Junyan Wu, Fangdi Hu, Yan Fang, Xuecai Li, Wancang Sun, Wangtian Wang, Chunsheng Bai

**Affiliations:** 1 State Key Laboratory of Aridland Crop Science, Gansu Agricultural University, Lanzhou, 7300070 China; 2 College of Agronomy, Gansu Agricultural University, Lanzhou, 730070 China

**Keywords:** Molecular biology, Plant sciences

## Abstract

Winter rapeseed (*Brassica rapa* L.) is a major oilseed crop in Northern China, where its production was severely affected by chilling and freezing stress. However, not much is known about the role of differentially accumulated proteins (DAPs) during the chilling and freezing stress. In this study, isobaric tag for relative and absolute quantification (iTRAQ) technology was performed to identify DAPs under freezing stress. To explore the molecular mechanisms of cold stress tolerance at the cellular and protein levels, the morphological and physiological differences in the shoot apical meristem (SAM) of two winter rapeseed varieties, Longyou 7 (cold-tolerant) and Lenox (cold-sensitive), were explored in field-grown plants. Compared to Lenox, Longyou 7 had a lower SAM height and higher collar diameter. The level of malondialdehyde (MDA) and indole-3-acetic acid (IAA) content was also decreased. Simultaneously, the soluble sugars (SS) content, superoxide dismutase (SOD) activity, peroxidase (POD) activity, soluble protein (SP) content, and collar diameter were increased in Longyou 7 as compared to Lenox. A total of 6330 proteins were identified. Among this, 98, 107, 183 and 111 DAPs were expressed in L7 CK/Le CK, L7 d/Le d, Le d/Le CK and L7 d/L7 CK, respectively. Quantitative real-time PCR (RT-qPCR) analysis of the coding genes for seventeen randomly selected DAPs was performed for validation. These DAPs were identified based on gene ontology enrichment analysis, which revealed that glutathione transferase activity, carbohydrate-binding, glutathione binding, metabolic process, and IAA response were closely associated with the cold stress response. In addition, some cold-induced proteins, such as glutathione S-transferase phi 2(GSTF2), might play an essential role during cold acclimation in the SAM of *Brassica rapa*. The present study provides valuable information on the involvement of DAPs during cold stress responses in *Brassica rapa* L, and hence could be used for breeding experiments.

## Introduction

Brassica winter rapeseed (*Brassica rapa* L.) is an effective surface cover oil crop that can survive the winters of northern China in the cold and arid region. Therefore, it has significant ecological and economic benefits in agricultural production^1^. However, the extremely low temperature in northern China negatively influences its regional distribution and production in winter^2^. Cold stress, including chilling (0–15 °C) and freezing (< 0 °C) temperatures, is one of the major limitations for the cultivation of Brassica winter rapeseed, as these conditions dramatically affect plant growth, yield, and oil quality^3,4^.

Previously, our group has used high-throughput sequencing technology to successfully identified cold-stress-responsive microRNAs (miRNAs)^5^, differentially expressed genes (DEGs)^6^ and differentially accumulated proteins (DAPs)^7^ in roots and leaves of *B. rapa*. Interestingly, we noted that the shoot apical meristems (SAM) of cold-sensitive rape were protruding, thus making them susceptible to environmental fluctuations, rendering the plants less able to successfully overwinter^8^. Conversely, the SAM of cold-tolerant rape was depressed, which likely afforded better buffering against the environment and improved hardiness. This suggests a direct role of the SAM in controlling cold tolerance^9^.

Further evidence on the role of SAM in cold tolerance is exemplified by the observations that the leaves of strongly cold-resistant plants grow more prone than their cold-sensitive counterparts^10^. Before winter, organic matter is preferentially distributed to the roots, storing enough organic matter and establishing an extensive root system, guaranteeing its safe overwintering and vegetativeness construction^11,12^. Growth regulation of the SAM is controlled partly by localized auxin biosynthesis and gradients and thus suggests a role of the hormone in cold tolerance^13^.

Given the apparent role of the SAM in conferring cold tolerance, the current study aimed to investigate differences at the morphological, physiological, and molecular levels localized within the SAM of two turnip rape genotypes divergent in cold tolerance. Specifically, a differential proteomics approach using an isobaric tag for relative and absolute quantification (iTRAQ) was employed^14^. This gel-free mass spectrometry technique has been successfully applied to identify differentially accumulated proteins involved in stress response across various plants such as *Vitis amurensis*^15^, *Triticum aestivum*^16^, *Gossypium hirsutum* L^17^, *Lycopersicon esculentum Mill*^18^, *Brassica rapa* L^19^*,* and *Spica Prunellae*^20^. Proteins showing localized differences in abundance can be targets for marker-assisted selection and used to improve breeding efficiency^14^. More importantly, this knowledge gives fundamental insight into the direct role of the SAM in controlling abiotic stress tolerance in plants.

The SAM depression and bulge in the seedling might be associated with their strong cold resistance. The functions of SAM target protein regulations have not yet been examined. Thus, the objective of the present study is to explore the relationship between SAM-target proteins regulations and cold stress tolerance. The proteins identified can be used to understand the molecular mechanisms underlying cold resistance. Proteins showing localized differences in abundance can be targets for marker-assisted selection, thus improving breeding efficiency. More importantly, this knowledge gives fundamental insight into the direct role of the SAM in controlling abiotic stress tolerance in plants.

## Results

### Physiological development and cold tolerance

**Figure 1:**
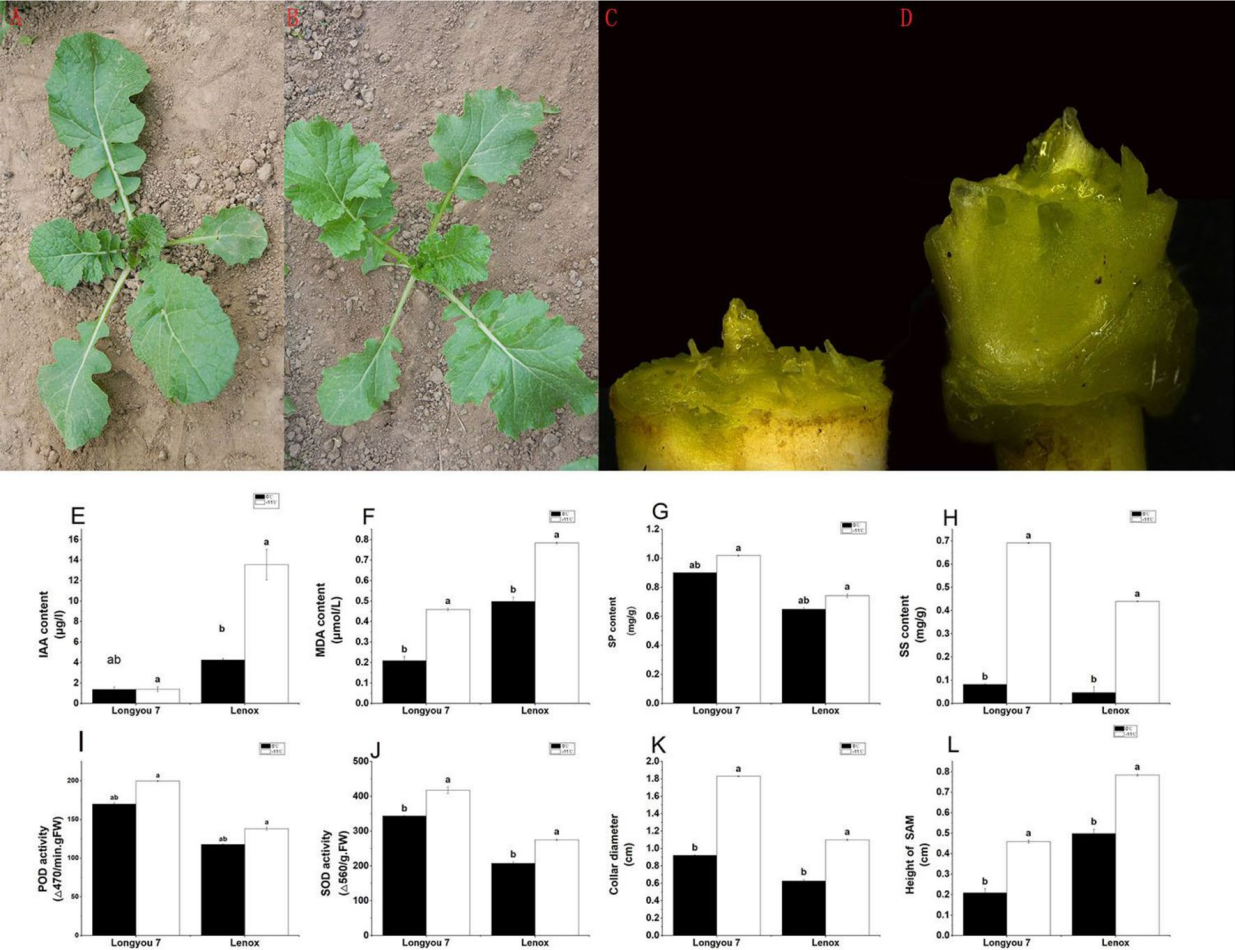
The growth characteristics of (**A**) Longyou 7 and (**B**) Lenox varieties. The SAM and collar morphology of (**C**) Longyou 7 and (**D**) Lenox. Longyou 7 and Lenox varieties (**E**) IAA content, (**F**) MDA content, (**G**) SP content, (**H**) SS content, (**I**) POD activity, (**J**) SOD activity, (**K**) Collar diameter, (**L**) Height of SAM. Error bars denote the standard error of the mean. An letter denotes significant differences between varieties at p ≤ 0.05. *SOD* superoxide, *POD* peroxidase, *IAA* indole-3-acetic acid, *SP* soluble protein, *MDA* malondialdehyde, *SS* soluble sugar.

The morphological and physiological differences between the cold-tolerant Longyou 7 and cold-sensitive Lenox were studied at the seedling stage under cold stress. Longyou 7 had a creeping petiole with leaves growing nearly prostrate (Fig. 1A), whereas Lenox had an upright petiole with leaf habit (Fig. 1B). When grown in an open field, the height of the shoot apical meristem (SAM) levels was measured at the different growth stages. A high SAM of Lenox was recorded at − 11 °C (d) compared to 0 °C (CK). Moreover, the height of the SAM of Lenox was significantly higher than Longyou 7 variety (Fig. 1C,D,L). Compared with the 0 °C, the collar diameter of Longyou 7 and Lenox was significantly increased during freezing stress at − 11 °C, and among both varieties, Longyou 7 showed higher collar diameter (Fig. 1C,D,K). Compared with the L7 d and Le d, the level of SOD and POD activity and SP and SS content significantly decreased in the L7 CK and Le CK samples (Fig. 1G–J). The MDA content significantly increased in L7 CK and Le CK. The IAA content of Le d was higher than that of L7 d (Fig. 1E). The MDA and content of Le CK and Le d were significantly higher than that of L7 CK and L7 d (Fig. 1F). These results suggested that Longyou 7 had smaller SAM but higher tolerance to freezing stress compared with Lenox. Strong cold-resistant varieties such as Longyou 7 were found to have a smaller SAM in the samples collected from field experiments, which were 5–10 cm below the soil surface. Thus, the SAM was located in sheltered moist soil under a relatively stable temperature, which is beneficial for winter *B. rapa* to the overwinter. These findings demonstrated an association between seedling SAM position and cold tolerance.

### iTRAQ-Based quantitative proteome analysis

Previous RNA-seq and iTRAQ-based quantitative proteome analysis applied on leaves and roots tissues were limited to obtaining relative differences in the genes or proteins expression under a controlled environment and did not account for the localized difference in the SAM. Our present study had demonstrated that DAPs were used to evaluate relative differences in protein expression as cold acclimation progressed under field conditions from early autumn to winter. The field trials were designed to capture the big picture of global changes of the proteomics during cold acclimation in Longyou 7 and Lenox varieties of *B. rapa*. The iTRAQ-based quantitative proteome analysis was performed in both varieties. The analysis of protein was performed using 8-plex iTRAQ-based comparative proteome analysis. Three independent biological replicates were simultaneously performed. After using Mascot to search against the *Brassica-rapa* 1.0 database, a total of 6 328 proteins were identified (Supplementary Table S1).

### iTRAQ quantitative identified DAPs

**Figure 2:**
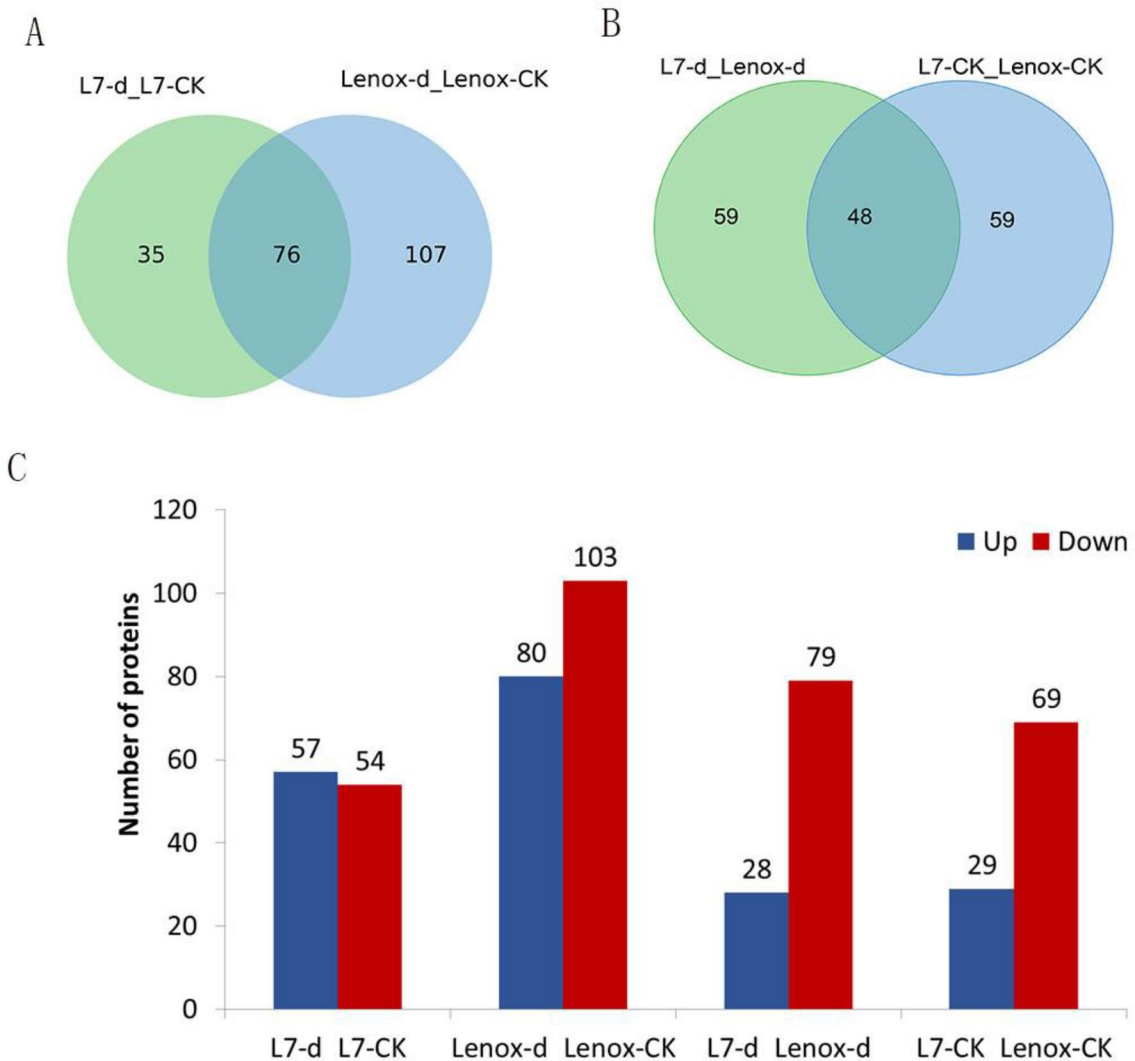
Venn diagrams of DAPs identified by iTRAQ between (**A**) L7 d/ L7 CK and Le d/ Le CK, (**B**) L7 d/ Le d and L7 CK/ Le CK. L7 d/ L7 CK is the protein abundance ratio of L7 d compared with L7 CK, Le d/ Le CK is the protein abundance ratio of Le d compared with Le CK, L7 CK/ Le CK is the protein abundance ratio of L7 CK compared with Le CK, and L7 d/ L7 CK is the protein abundance ratio of L7 d compared with L7 CK. (**C**) Number of up- and down-accumulated proteins among different comparison groups.

Based on fold change (FC) > 1.5 (p < 0.05), DAPs were up-accumulated, FC < 0.8 (p < 0.05), DAPs were down-accumulated. The Venn diagram reflected the DAPs in two winter rapeseed varieties. 76 DAPs were found between the Le d/Le CK and L7 d/L7 CK, and 48 DAPs were found in both the L7 d/Le d and L7 CK/Le CK (Fig. 2A,B). Of these, there were 35 DAPs unique to L7 d/L7 CK. As shown in Fig. 2C, 111 DAPs were expressed in L7 d/L7 CK with 57 up-accumulated and 54 down-accumulated DAPs (Supplementary Table S2). 98 DAPs were expressed in L7 CK/Le CK with 29 up-accumulated and 69 down-accumulated DAPs (Supplementary Table S3). 183 DAPs were expressed in Le d/Le CK with 80 up-accumulated and 103 down-accumulated (Supplementary Table S4). 107 DAPs were expressed in L7 d/Le d with 28 up-accumulated and 79 down-accumulated DAPs (Supplementary Table S5).

### Classification of cold responsive DAPs

**Figure 3:**
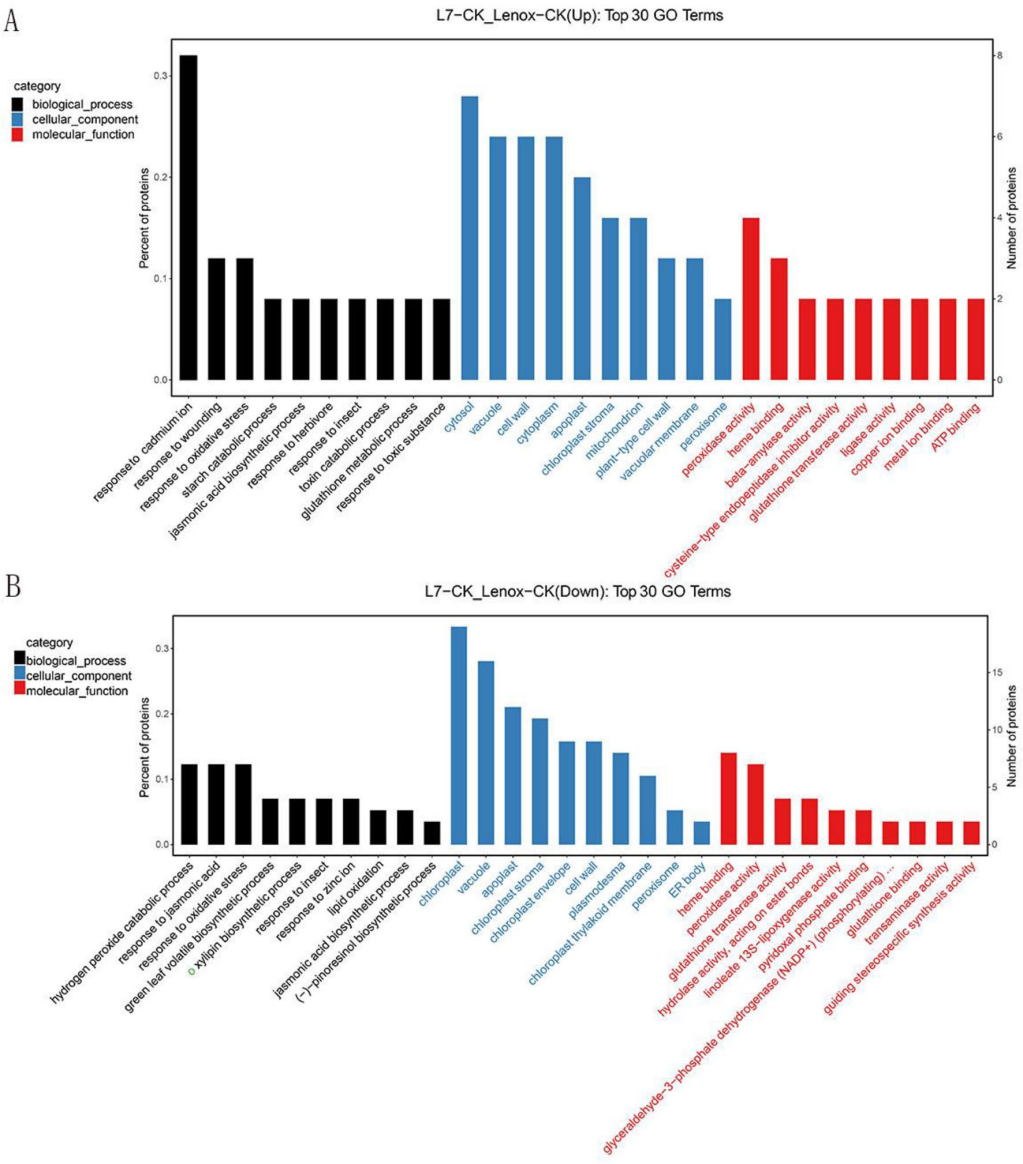
(**A**) Gene ontology (GO) enrichment analysis of up-regulated DAPs in L7 CK/Le CK. (**B**) GO enrichment analysis of down-regulated DAPs in L7 CK/Le CK.

To obtain the function of DAPs, all quantified proteins were searched through the UniProt-GOA database (https://www.ebi.ac.uk/GOA/) and gene ontology (GO) annotation (http:// www. panth erdb. org/). In total, 2669 identified proteins were annotated with GO terms. The top enriched GO terms of the DAP are shown in Fig. 3 and Supplementary Table S6. Cellular components, biological processes, and molecular function were assigned among DAPs based on the results of GO analysis. In L7d/Le d, most DAPs represented categories were hydrogen peroxide catabolic process, response to jasmonic acid and cadmium ion, and response to oxidative stress in biological_process. In addition, represented categories were chloroplast, cytosol, cell wall, vacuole, apoplast, and chloroplast stroma in cellular component; and heme-binding, peroxidase activity, and glutathione transferase activity in molecular function (Fig. 3 and Supplementary Table S6).

To further characterize the functions of the DAPs, KEGG pathway mapping was performed. KEGG enrichment analysis displayed the pathways of DAPs in L7 d/Le d. 24 DAPs were down-regulated in L7 d/Le d was found to be associated with glutathione metabolism (brp00480), thiamine metabolism (brp00730), and starch and sucrose metabolism (brp00500). Conversely, 6 DAPs up-regulated in L7 d/Le d were found to be associated with glutathione metabolism (brp00480), linoleic acid metabolism(brp00591), alpha-Linolenic acid metabolism brp(brp00592), pentose phosphate pathway(brp00030), phenylpropanoid biosynthesis(brp00940), and carotenoid biosynthesis(brp00906).

**Figure 4:**
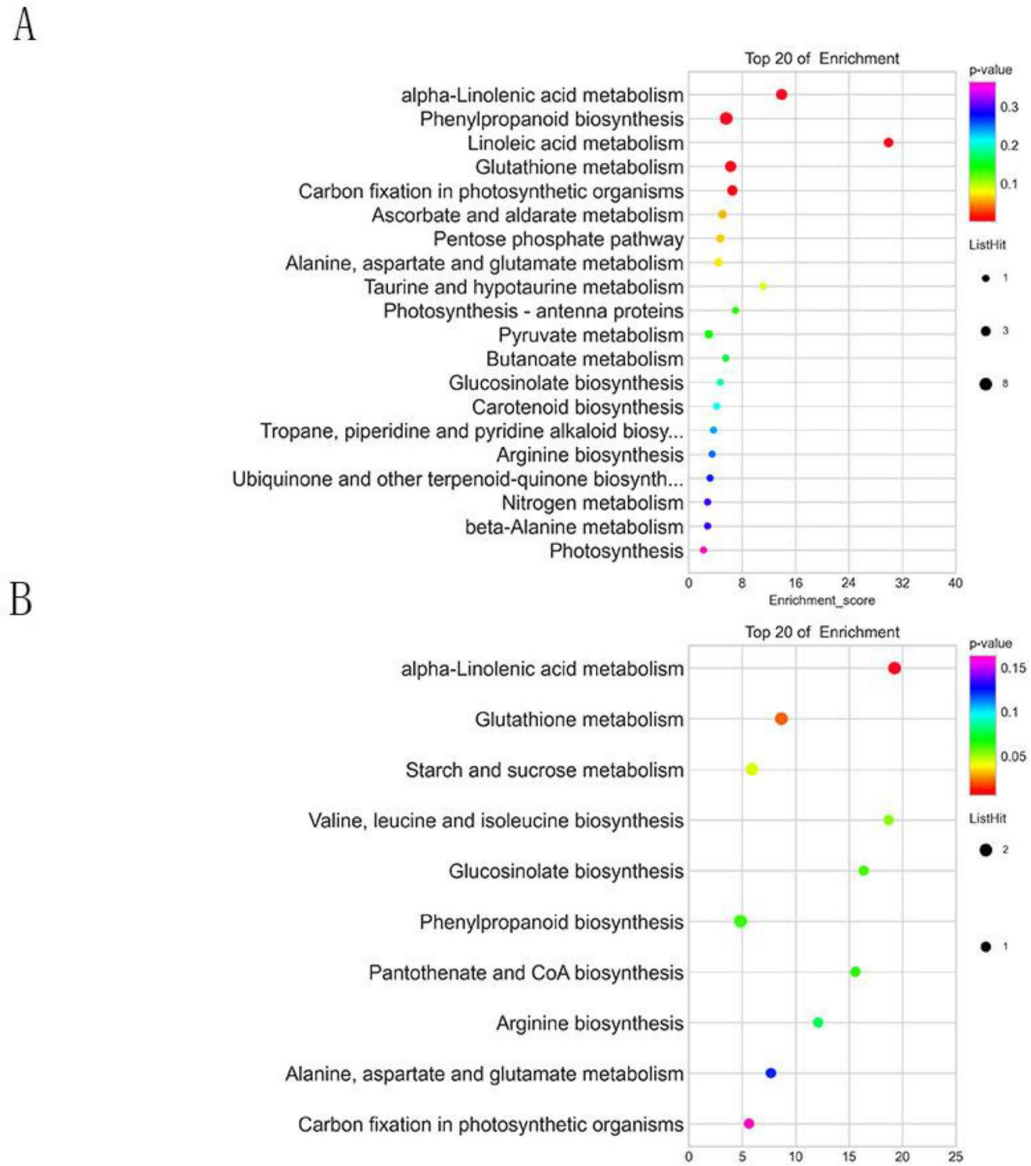
Kyoto Encyclopedia of Genes and Genomes (KEGG) pathway enrichment analysis of (**A**) down-regulated DAPs and (**B**) up-regulated DAPs in L7 d/Le d.

KEGG indicated that alpha-Linolenic acid metabolism(brp00592), phenylpropanoid biosynthesis(brp00940), linoleic acid metabolism(brp00591), glutathione metabolism(brp00480), and carbon fixation in photosynthetic organisms(brp00710) in down-regulated DAPs from L7 CK/Le CK. Moreover, alpha-Linolenic acid metabolism(brp00592), glutathione metabolism(brp00480), fatty acid degradation(brp00071), and starch and sucrose metabolism (brp00500) in up-regulated DAPs from L7 CK/Le CK. All the DAPs in KEGG categories are shown in Supplemental Tables S7 and Fig. 4.

### qRT-PCR revealed expression analysis of selected DAPs

**Figure 5:**
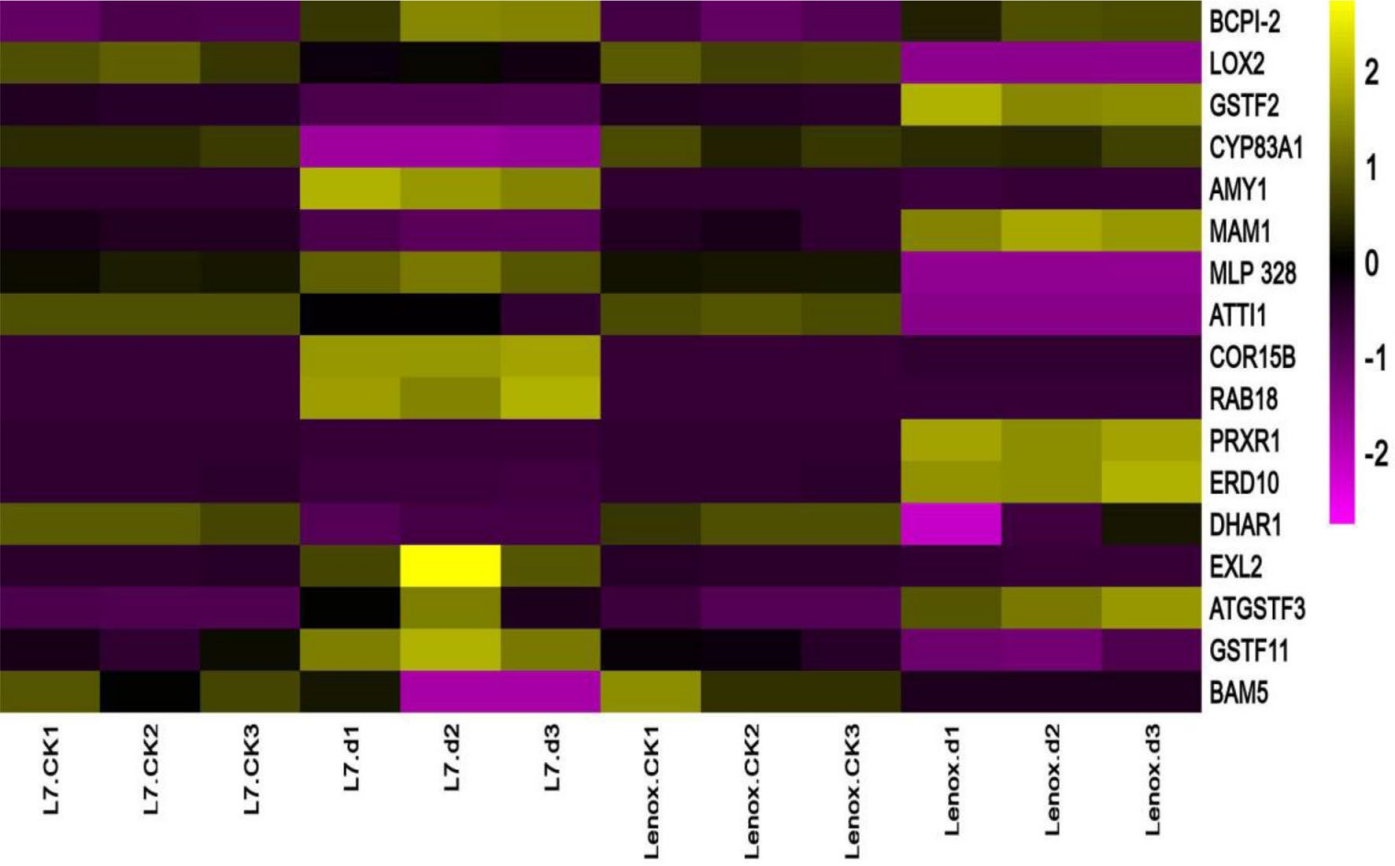
qRT-PCR analysis of genes related to DAPs. Transcript abundance was calculated according to the difference in cycle threshold values between the target gene and β-actin transcripts normalized by the 2 − ΔΔCT method.

Transcript expression levels were detected by qRT-PCR (Fig. 5). Seventeen genes were randomly selected to validate the reliability of the protein level data. Among those, seventeen genes displayed similar expression patterns to their protein levels in both varieties. These includes, BCPI-2(cysteine protease inhibitor, putative/cystatin, putative), LOX2 (lipoxygenase 2), GSTF2 (glutathione S-transferase 2), CYP83A1 (cytochrome P450 83A1), AMY1 (alpha-amylase-like), MAM1 (methylthioalkylmalate synthase 1), ATTI1(serine-type endopeptidase inhibitor), RAB18(responsive to ABA 18), PRXR1(peroxidase 1), DHAR1 (dehydroascorbate reductase), EXL2 (exordium like 2), ATGSTF3(glutathione S-transferase F3), ATGSTF11 (glutathione S-transferase F11), and BAM5 (beta-amylase 5). However, the expression of MLP 328(MLP-like protein 328) and ERD10 (early responsive to dehydration 10) was opposite with the protein abundances, probably due to various posttranslational modifications.

## Discussion

Proteomic analysis has identified many differentially accumulated proteins related to plant secondary metabolism in germinated seeds under cold stress^21^. In this study, a large number of cold stress-responsive proteins were identified in winter turnip rape. Previously several candidate genes had been identified by EST analysis which may be involved in cold stress response in winter turnip rape^5,6^. However, the mechanisms underlying the effect of cold stress on SAM are largely unknown. Therefore, physiological and proteomic analyses were performed in this study to examine the mechanism of the increased freezing tolerance in winter tunip rape at the SAM.

### Freezing stress affects morphology, physiological and biochemical changes in *Brassica rapa*

Freezing stress (< 0 °C) detrimentally affects plant growth and development, limits their geographic distribution, and significantly reduces agronomic productivity^22^. During the natural cooling process, plants change their morphological, physiological, and biochemical characteristics due to low-temperature stress, especially freezing^23^. The main target of freezing injury is the cell membranes rupture due to the expanding aqueous cellular content upon freezing. The lipids, peroxide, and MDA contents could reflect the damage of plants under low temperatures. The regulation of osmotic adjustment substances such as soluble protein and MDA could maintain the cellular structure and osmotic balance^24^. Meanwhile, MDA acts as a cell structure maintainer, a signaling molecule, and a ROS scavenger in response to cold stress. Among these, SOD and POD are the key antioxidant enzymes under stress^25,26^. These trends were observed in our data, where a decrease in the content of SS, IAA, MDA, and SP was observed in the control compared to the freezing stress treatment. Further, SOD and POD activity decreased in the CK compared to freezing stress (Fig. 1C) which is also in line with previous work^27–29^. Increased osmotic adjustment substance content could reduce the freezing point to increase plant cell membrane stabilization and protect membrane integrity during dehydration caused by freezing treatment in *B. rapa*. The winter rapeseed seedlings were damaged by low temperature. Among both the varieties, Longyou 7 showed strong antioxidant enzyme activity and high content of soluble regulators that prevent ROS-induced damage. These data showed that isometric and ROS were adjusted in *B. rapa* as a preventive measure against cold stress.

### DAPs are involved in lipid metabolism

Notably, our study found DAPs to be enriched in fatty acid metabolism functions. Several studies have shown that high concentrations of unsaturated fatty acids are commonly presented in plants grown under cold stress; thus, cold-tolerant plants often have higher unsaturated membrane lipids^30,31^. Linoleic and alpha-Linolenic acids play key roles in maintaining membrane integrity and fluidity and have been demonstrated to contribute to the osmotic tolerance in rice^32^. Our observation that enriched fatty acid metabolism-related proteins were differentially accumulated between the SAM of the cost-tolerant and cold-susceptible genotypes is therefore in agreement with published works. In a previous study, lipoxygenase (LOX) could damage cell membranes and other cell components. LOX2 was down-regulated stress and also played a key role in JA biosynthsis^33,34^. In this study, DAPs were involved in Linoleic acid, alpha-Linolenic acid, glycolipid, sphingolipid metabolism, and fatty acid degradation. Only fatty acid degradation was up-regulated, and the rest were down-regulated in L7 d/Le d. LOX2 (Bra004057, Bra003526, Bra003526) was down-regulated in L7 d/Le d, which is also in line with previous work^33^. It is known that LOX2 might play an important role in the cold stress of *B. rapa*.

### DAPs are involved in carbohydrate metabolism

Carbohydrate and energy metabolism are the basis of plant growth, development, and morphogenesis. Carbohydrate metabolism occupies the core of energy metabolism since it provides the essential saccharides and energy plants need. Starch and sucrose allocation is a well-regulated process in carbohydrate metabolism; especially, beta-amylase (BAM) is required for the starch breakdown in *A. thaliana*^35,36^. BAM was involved in the starch and sucrose metabolism^37,38^. It was reported that beta-amylase 5 (BAM5) might play an important role in the petunia response to cold stress^39^ and was up-regulated under heat stress^40^. The enzyme BAM accumulates SS under stress conditions^41^. Cold stress increased the content of soluble sugar and the activity of β-amylase^42^. Our study showed that the content of soluble sugar increased during cold stress, and BAM5(Bra038088) was down-regulated in L7 d/Le d and L7 CK/Le CK. This provides a new direction for future research in *B. rapa* under cold stress.

### DAPs involved in amino acid metabolism

The amino acid metabolic pathway was involved during cold/freezing stress in Arabidopsis, mycelia, loquat, and peach^43–46^. Glutamate decarboxylase (GAD) catalyzes the conversion of glutamate (Glu) to γ-aminobutyric acid (GABA)^47^. GABA plays multiple roles in plant stress adaptation and ROS scavenging^48^. Thus, glutamate played an important role in amino acid metabolism^49^. Glutamate decarboxylase isoenzymes (GAD2) played a negative role in tolerance to freezing stress^50^. In the present study, GAD2(Bra004136, Bra004137) has low log2 FC in L7 d/Le d and L7 CK/Le CK, which is in line with previous work^50^. Therefore, GAD2 might play some essential roles in response to the cold stress in the *B. rapa* under cold stress.

### DAPs involved in translation

Posttranslational modification (PTM) in *Brassica juncea* cold stress systems include S-nitrosylation^51^ and confers the glutathione S-transferase (GST) under oxidative stress^52^. GSTs are a major family of detoxification enzymes that protect plants against oxidative damage^53^. Auxin induced the expression of several GSTs such as GSTF2, which get up-regulated and might be linked with stress-mediated growth responses^52^. It was found that GSTF2 enhanced the tolerance to cold stress in wheat^53^. Our study showed that higher SAM height indicated relatively high levels of IAA content, while a relatively smaller SAM indicated relatively low levels of IAA content; hence, auxin content is profoundly related to the SAM height. Also, five GSTs (Bra000875, Bra021673, Bra000876, Bra036259, and Bra012409) and DHAR 1 (Bra025749) were down-regulated, while GSTU (Bra012422, Bra000474) and GSTs (Bra032010) were up-regulated in L7 d/Le d. The increased accumulation of these proteins indicated that plant cells initiated their antioxidant mechanisms to maintain redox homeostasis and resist cold stresses. Also, GST was involved in posttranslational modification in L7 d/ Le d and L7 CK/Le CK. It played a crucial role in improving the genetics of low-temperature tolerance in plants.

## Conclusion

In this study, two winter different winter turnip rapa cultivars (Longyou 7 and Lenox) of *Brassica rapa* L. with different phenotypes for cold stress were used to analyze molecular mechanisms for the cold response. Based on the proteomics data, it was found that major proteins were involved in metabolic pathways through differentially accumulated proteins (DAPs) in both winter turnip rape varieties (Longyou 7 and Lenox) under cold stress. In addition, based on the functional analysis, we concluded that DAPs involved in amino acid, carbohydrate, lipid, and energy metabolism, which showed that DAPs greatly changed freezing stress of winter turnip rape. In summary, these findings improve our understanding on the molecular mechanism of freezing tolerance, which helps in breeding winter turnip rape against the cold stress.

## Methods

### Plant materials, field, sample collection

Two different winter turnip rapa cultivars were used in this study. Longyou 7 is a cold-tolerant variety, and Lenox is a cold-sensitive variety; both were collected from Gansu Agricultural University (Gansu, China). Plants were grown and maintained following local best agronomic practices. Seeds were sown in the field on August 20, 2018, and the seedlings emerged on August 27. The experiment was conducted in a normal agronomic field trial at the Gansu Research Center of Rapaseed Engineering and Technology in Lanzhou, China. Three rows of Longyou 7 and Lenox, respectively, were planted. Each row was 2 m long, with a row spacing of 20 cm and plant spacing of 10 cm under manual spot seeding. Seedling emerged within 7–10 days after sowing. Three replicates were prepared for each cultivar in the field. Samples were harvested on October 13 (five true leaves stage) as control (CK). Samples were taken on December 16 (seven true leaves stage) as a treatment (d). Measurements of the height of SAM and collar diameter were subsequently taken on October 13 and December 16, 2018.

Data were collected daily at average daily minimum temperature (Tmin) from October to December. The Tmin was 0 °C On October 13 and − 11 °C on December 16. The temperature of October 13 and December 16 is the mean value of the lowest temperature in 15 days. The samples for analysis were named, Le CK (sample of “Lenox” at 0 °C October 13), Le d (SAM of “Lenox” at − 11 °C), L7 CK (SAM of “Longyou 7” at 0 °C), and L7 d (SAM of “Longyou 7” at − 11 °C). SAM (shoot) was collected from four to six plants in each point and replicates. Plants were collected in the field between 10:30 am and 11:30 am to minimize the circadian rhythm effect for each sampling point. The SAM (less than a 1 cm long section at the crown base, Fig. 2B) was used periodically for physiological analyses. SAM was flash-frozen in liquid nitrogen and stored in a freezer at − 80 °C. SAM was used for conducting physiological analyses, RNA extraction, and iTRAQ analysis.

### Morphological and physiological analyses

Height of SAM and collar diameter were determined using a vernier caliper. The samples were analyzed in triplicates. POD activity^54^, CAT activity^54^, SS content^55^, IAA content^56^, SP and MDA content^57^ were analyzed as indicators of physiological response. The majuscules indicated a significant difference (p < 0.05) for the freezing stress-treated samples compared with chilling stressed samples. Values are the means and SD of three biological replicates, each calculated from the mean of three technical replicates.

### Protein extraction

Three biological replicates were used for iTRAQ-based comparative proteomics analysis. Approximately 500 mg from each biological replicate was ground into powder using liquid nitrogen and dispersed in (vortex blending) 500 µL extraction buffer (0.7 M sucrose, 0.1 M NaCl, 0.5 M Tris–HCl (pH 7.5), 50 mM EDTA and 0.2% DTT). The samples were homogenized at 60 Hz for 2 min. Then the extraction buffer (1 mL) was added and mixed, followed by adding Tris-phenol buffer and mixing for 30 min at 4 °C. The homogenates were centrifuged at 7100×*g* for 10 min at 4 °C. The supernatant was collected, added to five 0.1 M cold ammonium acetate methanol buffer volumes, and stored at − 20 °C overnight.

The homogenates were centrifuged at 12,000×*g* for 10 min to collect precipitations. The methanol was replaced with acetone, and the steps used to obtain precipitate were repeated. Next, the samples were centrifuged at 12,000×*g* for 10 min at 4 °C to collect supernatants. The supernatants were dried at room temperature for 5 min and dissolved in sample lysate form 2 h. The supernatants were centrifuged again to remove precipitations completely. The protein concentration was quantified using a commercial bicinchoninic acid assay following the manufacturer’s protocol (Thermo Fisher Scientific). The assay was performed in a microwell plate with three technical replicates for each sample against a bovine serum albumin standard curve.

### Protein digestion, iTRAQ labeling, and RP chromatography separation

For proteomic analysis, 15 µg of protein from each sample was separated on a 12% polyacrylamide gel. Protein digestion was performed according to the FASP procedure^58^. IAA was added to the final concentration of 50 mM in the dark for 40 min. The protein solutions were centrifuged at 12,000×*g* for 20 min at 4 °C. The supernatant was collected, and 100 mM tetraethylammonium bromide (TEAB, pH 8.0) was added before centrifuging at 12,000×*g* for 20 min. The solutions were collected and lyophilized. The lyophilized samples were suspended in TEAB (100 μL, 50 mM), and 40 μL of each sample was transferred into new tubes for iTRAQ labeling. The iTRAQ labeling reagent (iTRAQ® Reagents-8plex kit, Sigma) was used following the manufacturer’s protocol (Applied Biosystems, Foster City, CA, USA). All labeled peptides were pooled together. Subsequent analyses were performed at the Shanghai Luming biology center (Shanghai, China). iTRAQ labeled peptides were fractionated by RP chromatography using the 1100 HPLC System (Agilent). RP separation was performed on the Agilent Zorbax Extend RP column (5 μm, 150 mm × 2.1 mm). Tryptic peptides were separated at an eluent flow rate of 300μL min^−1^ and monitored at 210 and 280 nm. Dried samples were harvested from 8 to 50 min, and elution buffer was collected every minute, numbered from 1 to 10 chronologically. The separated peptides were lyophilized for MS detection^59^. The protein concentration was quantified using the BCA method, and the protein purity was detected by SDS-PAGE^60^.

### Mass spectrometry analysis

Compared with MS, LC–MS analyses were performed on a Q-Exactive mass spectrometer (Thermo, USA) equipped with a Nanospray Flex source (Thermo, USA). The peptides mixtures were loaded by a capillary C18 trap column (3 cm × 100 μm, C18, 3 μm, 150 A) and separated by a C18 column (15 cm × 75 μm, C18, 3 μm, 120 A) on a ChromXP Eksigent system (AB Sciex). Full MS scans were acquired in the mass range of 300–1600 mass compared with a mass resolution of 70,000, and the AGC target value was set at 1,000,000. The 10 most intense peaks in MS were fragmented with higher-energy collisional dissociation (HCD) with a collision energy of 30%. MS spectra were obtained with a resolution of 17,500 with an AGC target of 200,000 and a max injection time of 50 ms. The Q-E dynamic exclusion was set for 15.0 s and run under positive mode^61^.

### Protein identification and function annotation

Raw data of iTRAQ-labeled proteins were compared against the *Brassica rapa* genome protein database at the National Center for Biotechnology Information (NCBI) using the Proteome Discoverer TM 2.2 (Thermo, USA). Database searches were performed with trypsin digestion specificity, and the cysteine alkylation option was applied. For the protein quantification method, the iTRAQ 8-plex was chosen. A decoy database search approach was used for protein identification to determine the false discovery rate (FDR) with an acceptance threshold of FDR < 1.0%, while protein identification required at least two peptides. The molecular functions of the identified proteins were classified according to their gene ontology annotations and their biological functions. Only the proteins identified with at least two different peptides (*p* < 0.05), and quantified with a ratio of fold change (FC) > 1.5 or FC < 0.8 were considered. The NCBI (https://www.ncbi.nlm.nih.gov/) and Uniprot databases (https://www.uniprot.org/) were chosen for the validation and annotation of the protein sequences. Gene Ontology (GO) annotation for the identified proteins was assigned to the Uniprot database (http://geneontology.org/). The Kyoto Encyclopedia of Genes and Genomes (KEGG) (http: www.kegg.jp/kegg/kegg1.html) was used to predict the main metabolic pathways^62^.

### RNA extraction and qPCR analysis of gene expression

Three biological replicates and three technical replicates were performed for each gene. According to the manufacturer's instructions, the total RNA from the SAM was extracted by using TRNzol Universal Reagent (Tiangen, China). 1 μg of total RNA from each sample was used for first-strand cDNA synthesis according to the protocol supplied with PrimeScript™ RT Master Mix (TaKaRa Biotechnology Dalian, China). Genes for analysis were selected, and primers were designed using Primer-BLAST (https://blast.ncbi.nlm.nih.gov/Blast.cgi) (Bethesda, MD, USA) based on the sequences of the selected indigenes. Actin was selected as reference genes. qRT-PCR amplification reactions were performed using a LightCycler®96 Real-Time PCR System (Roche, Basel, Switzerland). Genes were assayed in technical triplicates for each sample. The relative gene expression was calculated using the 2 − ∆∆Ct method^63^. The primer sequences were designed by Primer-BLAST (https://blast.ncbi.nlm.nih.gov/Blast.cgi) (Bethesda, MD, USA) based on the sequences of the selected indigenes (Supplementary Table S8). Mean values and standard errors were calculated from three independent experiments with three biological replicates, and relative expression was calculated using Actin's 2 − ∆∆Ct method as the reference.

### Statistical analysis

The control and treatment groups were analyzed for statistical significance of differences between multiple groups using one-way analysis of variance (ANOVA) followed by Duncan’s multiple comparisons test. All calculations were performed using SPSS software (version 19.0, IBM, Armonk, NY, USA). All results are presented as mean ± standard deviation from three independent biological replications. Treatment means were separated by the Duncan multiple range test at P < 0.01. We used the minmax normalization method through the R programming language to analyze transcriptional and proteomic data and presented expression values using heat maps.

## Supplementary Information

Supplementary Table S1.

Supplementary Table S2.

Supplementary Table S3.

Supplementary Table S4.

Supplementary Table S5.

Supplementary Table S6.

Supplementary Table S7.

Supplementary Table S8.
