# Author Correction: iTRAQ-based quantitative proteome analysis insights into cold stress of Winter Rapeseed (*Brassica rapa* L.) grown in the field

**DOI:** 10.1038/s41598-022-07503-x

**Published:** 2022-02-28

**Authors:** Zaoxia Niu, Lijun Liu, Yuanyuan Pu, Li Ma, Junyan Wu, Fangdi Hu, Yan Fang, Xuecai Li, Wancang Sun, Wangtian Wang, Chunsheng Bai

**Affiliations:** 1 State Key Laboratory of Aridland Crop Science, Gansu Agricultural University, Lanzhou, 7300070 China; 2 College of Agronomy, Gansu Agricultural University, Lanzhou, 730070 China

Correction to: *Scientific Reports* 10.1038/s41598-021-02707-z, published online 06 December 2021

The original version of this Article contained an error in the Funding section. Furthermore, the Acknowledgements section was a duplicate of the Funding section. The duplicate has been removed in the original Article.

The Funding section

“Funding was funded by National Natural Science Foundation of China (Grants NO. 31960435, NO. 31860383), Research Program Sponsored by State Key Laboratory of Aridland Crop Science (Gansu Agricultural University), GSCS-2020-06, China Agriculture Research System of MOF and MARA, CARS-12, Research Program Sponsored by State Key Laboratory of Aridland Crop Science, Gansu Agricultural University, No. GSCS-2020-Z1.”

now reads:

“Funding was financially supported Research Program Sponsored by State Key Laboratory of Aridland Crop Science, Gansu Agricultural University (No. GSCS-2020-Z1); by the China Agriculture Research System of MOF and MARA (CARS-12); by the National Natural Science Foundation of China (NO. 31960435); by the Research Program Sponsored by State Key Laboratory of Aridland Crop Science (Gansu Agricultural University) (GSCS-2020-06) and the National Natural Science Foundation of China (NO. 31860388).”

The original Article has been corrected.

