# Supplementary material for: iTRAQ-based quantitative proteome analysis insights into cold stress of Winter Rapeseed (Brassica rapa L.) grown in the field
Source: Sci Rep. 2021 Dec 6;11:23434. doi: 10.1038/s41598-021-02707-z (PMC8648733; doi:10.1038/s41598-021-02707-z)
Supplement: Supplementary file 8 — Supplementary Table S8. [file 41598_2021_2707_MOESM8_ESM.docx]

Table S8 Primers for qRT-PCR analysis of genes of differentially expressed proteins

| Uniprot ID | Protein name | Primer Sequences （5 '-3'） | |
| --- | --- | --- | --- |
|  |  | Primer F | Primer R |
| GSTF2 | Glutathione s-transferase 2 | CAAGGACGGTGAGCACAAGACAG | CTTCGGCGACAACGGCTTGG |
| BAM5 | Beta-amylase 5 | CCGTTACCCTGCTTACCCACAAAG | GCCGCTGCTTCCTTGAACTCG |
| DHAR 1 | Dehydroascorbate reductase 1 | CTCCTCCCAAGTTTGCCTCTGTTG | GCCCATCCTGCGATCACATACTTC |
| GSTF11 | Glutathione s-transferase 11 | AGCCGTTTGGTCAAGTTCCA | TTGCCCAATAGGTCCGTTCC |
| MAM1 | Methythioalky malate synthase1 | TGCTACCGCCAACACAATCGC | CACCACCCATCACAAATGCTCCTC |
| PRXR1 | Peroxidase 1 | CCGCTTTCTCTTGGCTCCGTAAC | GCTCCTGTCGTGTTCTTTCTCTCC |
| ATTI1 | Serine-type endopeptidase inhibitor | TCAAGGACGGTGAACACAAGAAAGAG | CGGTGAGCTATGTACTGAGTAATGGC |
| CP450 83A1 | Cytochrome P450 83A1 | CATCATCGGCGTGGTGACTATCG | GGTTCTGAAGCTGGTGGAGGTTTC |
| EXL2 | Exordium like 2 | TACGGAACTAACACGGCTGCTTTG | AGTTGGTTGCGGTAATGAGGATCG |
| BCPI-2 | Cysteine proteinase inhibitor 12 | AGATCGTCGAGGCTGGGAAGAAG | CACCATCAGAGGCAGGCTTGAAC |
| ERD10 | Early reponsive to dehydration 10 | TGCGACGACGGGAGAGGTTAAG | TCCGAGACCTGAGCCTTGTGC |
| COR15B | Cold regulated 15B | TTCTTCTTTCTCCAGCGGCATAGC | TCGGCGTAGATCAACGACTTCTTG |
| RAB18 | Responsive to ABA 18 | TTACGGGACAGGTGGCGAAGG | GATCCAGAGCGGTGAAGCATTCC |
| MLP328 | MLP-like protein 328 | TACAAGAGGTGGAGGAGCGAGAAC | CCATCGCATGTGTAGTCCCAAGTC |
| DHAR1 | Ehydroascorbate reductase 1 | CAAAGACTCCAGCGACGGAT | CAAGCTCTCGGGAACAGACC |
| AMY1 | Alpha-amylase-like 1 | ACAGCTCCAAGTACGGTTCG | CTCTCAGCCGTTCTGTGGTT |
| LOX2 | Lipoxygenase 2 | CGTTGAGCTTATTTCCGCCG | GCGAGCAAATTCGTCGTCTC |

| **Gene Name** | **Forward (5'→3')** | **Reverse (5'→3')** |
| --- | --- | --- |
| *β-actin* | GACTGGTATGGGTGGTTT | GGTAGCACAAGCGGTAGA |
